# Speckle-Free, Angle-Free, Cavity-Free White Laser with a High Color Rendering Index

**DOI:** 10.1021/acsami.3c17222

**Published:** 2024-02-23

**Authors:** Cheng-Fu Hou, Wei-An Tsui, Rou-Jun Chou, Chih-Hao Hsu, Denice N. Feria, Tai-Yuan Lin, Yang-Fang Chen

**Affiliations:** † Department of Physics, National Taiwan University, Taipei 10617, Taiwan; ‡ Department of Optoelectronics and Materials Technology, National Taiwan Ocean University, Keelung City 202301, Taiwan

**Keywords:** angle free, speckle free, white random laser, multiple scattering, color rendering index

## Abstract

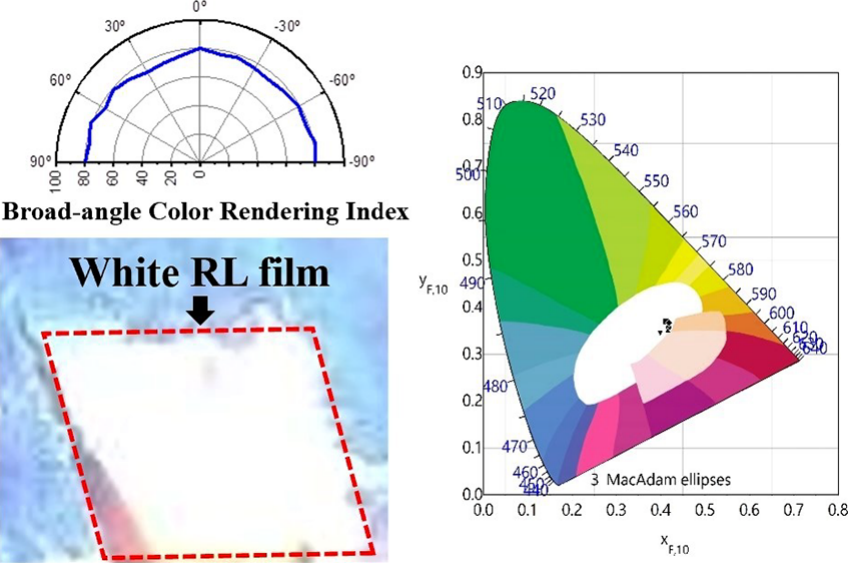

The freedom from efficiency droop motivates monochromatic lasers to progress in general lighting applications due to the demand for more efficient and sustainable light sources. Still, a white light based on monochromatic lasers with high lighting quality, such as a high color rendering ability, an angle-independent output, and a speckle-free illumination, has not yet been fabricated nor demonstrated. Random lasers, with the special mechanism caused by multiple scattering, the angle-free emission, and the uncomplicated fabrication processes, inspire us to investigate the feasibility of utilizing them in general lighting. In this work, a white random laser with a high color rendering index (CRI) value, regardless of pumping energy and observing direction, was performed and discussed. We also investigated the stability of white RL as its CIE chromaticity coordinates exhibit negligible differences with increasing pump energy density, retaining its high-CRI measurement. Also, it exhibits angle-independent emission while having a high color rendering ability. After passing through a scattering film, it generated no speckles compared to the conventional laser. We demonstrated the advances in white laser illumination, showing that a white random laser is promising to be applied for high-brightness illumination, biological-friendly lighting, accurate color selections, and medical sensing.

## 1 Introduction

Laser ignites the possible portrait of the general lighting in the future. With the more significant efficiency droop reduction, higher output irradiance, and smaller required chip area, lasers have enormous potential to match or even replace light-emitting diodes (LEDs) for a critical role in smart-lighting and sustainable light laser modules or prototypes have been extensively conducted. Simultaneous red, green, and blue (RGB) lasers have been extensively conducted, including the white laser consisting of a flute-type hollow-cathode He–Cd+ laser tube and a broadband optical cavity,^1^ the Ar–Kr mixed gas laser,^2^ the monolithic multisegment ZnCdSSe-based semiconductor nanosheet white laser,^3^ a simultaneous RGB laser based on a single-chip dye-doped polymer device,^4^ and so on. However, due to the natural coherence of the white laser, before wild application in general lighting, there remains a long way to go for several significant challenges, such as high directivity and laser speckle. The low divergence of lasers is double-edged, making laser a focused radiation source rather than an ideal light source uniformly across a complete 4π solid angle. The high spatial coherence of lasers also results in laser speckles, blurring the images under the illumination of laser lighting. Although methods to reduce the defects have been studied, much bulky, costly, and intricate designs are inevitably needed. While the color difference between RGB/RYGB lasers and general white light sources can be indistinguishable for human eyes, the color rendering index (CRI) is considered to be the indicator of illumination quality. However, there is still less evidence of a promising CRI of these RGB/RYGB lasers.

In the highly industrialized world, an ideal light source should fulfill the goal of illuminating and decorating the living space and offering natural and healthy lighting. Several studies have linked the relationship between the excellent quality of light to the physiological mechanism of the human being, such as healing depression, preventing breast cancer indirectly caused by melatonin (MLT) suppression, improving sleep-wake rhythm disturbances with Alzheimer’s disease, and so on.^5−8^ Though the mechanism of these biological effects of light quality still requires further studies, some researchers point out that a bright, full-spectrum, and biologically friendly white light should be designed and applied in indoor and outdoor lighting for a healthier life.^9^ To realize and manufacture these high-quality and continuous-spectrum white lights competently, it is worth mentioning that these white lights have features including better visibility, circadian-friendliness, and, last but not least, a high color rendering ability. Color rendering is the ability of a light source to faithfully grant objects the right colors compared with an ideal light source, and currently, the CRI, also known as Ra, defined by the International Commission on Illumination (CIE), is the common qualified index. Several studies have shown that human eyes are relatively more sensitive to the slightly unnatural color of images than to the difference in light intensity since there are few mechanisms for the visual system to adjust abnormal color, unlike the one called “chromatic adaptation” for change in illumination, extensively stressing the importance of CRI.^10,11^ Also, it has been demonstrated that a light source with a high CRI offers preferable visibility and that color distortion under a poor CRI lighting source can cause discomfort.^12−14^ Nevertheless, the color rendering ability of RGB laser has not yet been promised owing to its narrow line width hindering RGB laser from filling the entire visible spectrum and securing the light quality. Deficiencies of conventional monochromatic lasers in lighting quality and color rendering ability motivate us to discover another solution for the laser to fit the demand for healthy lighting.

Random laser, first theoretically proposed by Letokhov in 1968 and experimentally demonstrated afterward, provides a possible answer to solve the shortcomings of the conventional laser.^15^ The random lasing system used multiple scattering inside disordered materials instead of mirrors to form closed loops that trapped light and induced amplified feedback. With this unique feedback mechanism caused by multiple scattering, spiky stimulated peaks based on the feedback mechanism determined by scattering, the mean free path could be found in the spectrum when about the threshold, also known as one of the distinguishing features of random lasers.^16^ Furthermore, random lasers show a highly irregular spatial mode due to the random-walk propagation of light in the scattering materials, proving that different scattering strengths and pump geometry can attain a lower coherence. Due to the unconventional method of forming random lasers, high spatial coherence behavior is unnecessary for a random laser, which is a significant merit for breaking into the general lighting market space. Without high spatial coherence, the random laser can undoubtedly overcome the speckle effect, and the application of speckle-free imaging has been demonstrated.^17^ Besides, the output of the random laser can be observed in different directions by the various lasing modes of multiple scattering, which leads random lasers to an almost ideal angle-free light source.^18−21^ These two properties liberate lasers from any complicated design to offer speckle-free and angle-free light, and the emergence of the white random laser (white RL) has just laid the foundations for achieving the goal of general lighting. Such an unconventional laser system can be realized in soft materials and limitless innovative applications such as soluble, stretchable, and inkjet-printed random lasers.^22−25^

In this work, the light quality and CRI of the white RL, the critical piece of the missing puzzle for laser general lighting application, have been investigated and discussed. Based on Grassmann’s law, various colors can be tuned within the gamut spanned by the lighting source by mixing fundamental colors of lasers. Thus, to thoroughly study the color rendering ability of white RL, we created the composite spectra of the white RL by RYGB spectra from four organic lasers light-pumped under different energies and observed from various angles. Last but not least, the speckle-free images under the illumination of the white RL have been reproduced. Our experiment result brought a new realization of proving the feasibility of the random laser. This new observation breaks into the arena of general lighting and examines that a white RL can render distinct objects irrespective of pumping energy and observing angle. With a continuous and biology-friendly spectrum, speckle-free illumination, and the high light quality mentioned earlier, the white RL can meet the standard of an ideal lighting source. Furthermore, several features of organic materials of a random laser, such as low cost, flexibility, convenience, etc., lighten up the colorful applications. The white RL is likely to become one of the possible keys that could improve the disadvantages of conventional monochromatic lasers and shine the future for healthy, high-lumen, and high color rendering laser lighting.

## 2 Materials and Methods

### 2.1 Sample Synthesis

The glass substrates (1.5 × 1.5 cm) were ultrasonically cleaned subsequently for 10 min in deionized (DI) water, acetone, and isopropyl alcohol (IPA) in sequence to remove any adsorbed contaminant.

#### 2.1.1 Solution-Based Silver Nanoparticle Synthesis

0.5 mg of silver nitrate (AgNO_3_) and 150 mg of poly(vinyl alcohol) (PVA) were evenly dissolved in 5 mL of DI water. Then, the mixture was heated at 120 °C for 6 h. Finally, with this in situ reduction process, silver nanoparticles were naturally formed in this precursor solution.

#### 2.1.2 Red Monochromatic Polymer Films

4-(Dicyanomethylene)-2-*tert*-butyl-6-(1,1,7,7-tetramethyljulolid-in-4-yl-vinyl)-4H-pyra (DCJTB) was dissolved in dichloromethane (DCM) with a concentration of 10 mg/mL at room temperature. The DCJTB solution was directly jetted on the glass substrates. Under annealing at 100 °C for 10 min, self-assembled DCJTB nanocrystals were formed. Next, poly(methyl methacrylate) (PMMA) was spin-coated onto the DCJTB layer at a rate of 6000 rpm for 20 s to protect the nanostructures and heated at 100 °C for 10 min to dry out the PMMA. As a result, free-standing red monochromatic polymer films (red-MPFs) were formed on the glass substrates.

#### 2.1.3 Yellow-MFPs

Rhodamine 6G was dissolved in precursor solution with the ratio of 0.25 mg: 1 g. The solution was directly dropped on the glass substrates and heated at 120 °C for 10 min.

#### 2.1.4 Green-MFPs

Rhodamine 110 was dissolved in methanol with the ratio of 1 mg:1 g by weight. Next, the dye solution and precursor solution were mixed with a ratio of 1:5 by weight. The solution was directly dropped on the glass substrates and heated at 120 °C for 10 min.

#### 2.1.5 Blue-MFPs

Stilbene 420 was dissolved in ethanol with the ratio of 3 mg:1 g. Next, the dye solution and precursor solution were mixed with the same ratio by weight. The solution was directly dropped on the glass substrates and heated at 100 °C for 10 min.

### 2.2 Optical Measurements

The samples were optically excited by frequency-quadrupled 266 nm pulsed Nd:YAG laser (NewWave, Tempest 300) with 4 ns pulse width and 10 Hz repetition. The pumping beam was focused by a spherical lens (*f* = 100 mm). A bandpass filter of 20 nm width was used to block the pump laser illumination. The emission properties were spectrally analyzed using a high-resolution spectrometer Jobin Yvon iHR550 with gratings of 300 (spectral resolution 0.15 nm) and 1200 grooves/mm (spectral resolution 0.0375 nm). A synapse thermoelectric cooled charge-coupled device (CCD) guaranteed to −75 °C was connected to the spectroscopy software SynerJY. Figure S4 shows the schematic representation of the experimental setup for the images of the AF chart measured by conventional laser and random laser light sources composed of an optical microscope, optical fiber, the CCD, and the 266 nm light source.

## 3 Results and Discussion

**Figure 1:**
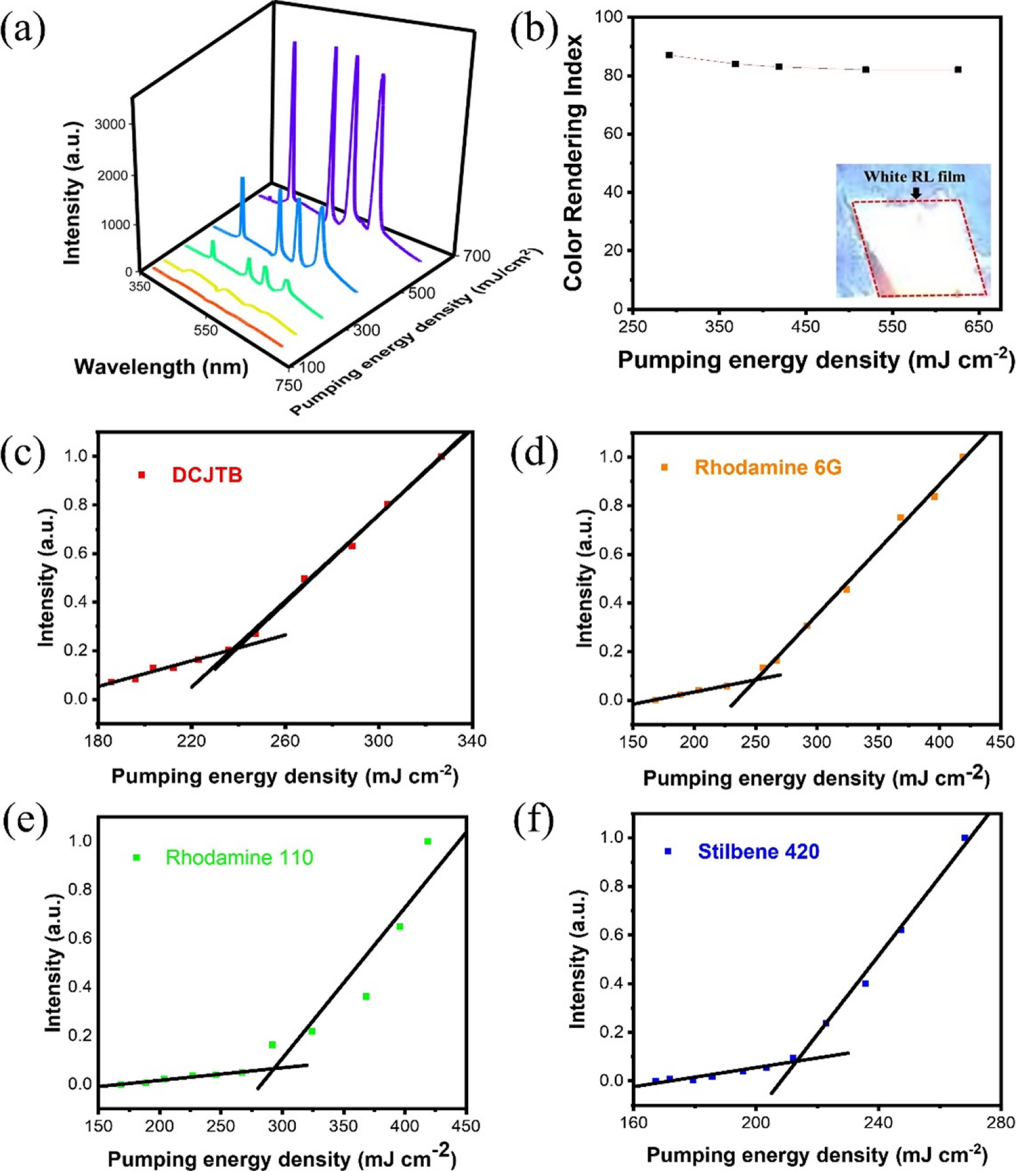
(a) White RL emission spectra, (b) CRI at different pumping energy densities of the white RL (inset: photograph of the white RL film), and (c–f) threshold of RYGB random laser.

Ideally, a light source should provide an acceptable chromaticity and a CRI, regardless of output intensity. Therefore, for a random laser light source, it is necessary not to distort the colors of the living space both below and above the threshold, that is, with the illumination under both spontaneous emission and stimulated emission. To study the color rendering ability of the white RL under different pumping energies, the samples are optically pumped by a 266 nm pulsed laser. Figure 1 shows the emission spectra, CRI, and emission intensities at different pumping energy densities of the white RL. The emission spectra as the function of pumping energy density are shown in Figure 1a, providing the characteristics of the white RL. Broadband peaks of spontaneous emission were observed in the spectrum at low pumping densities of 160 to 250 mJ/cm^2^. While the power density increased near the threshold, amplifying the light trap in the closed loop more than the loss, stimulated emission results in multiple peaks, which emerge at approximately 650, 575, 540, and 440 nm, representing RYGB light color. As pumping power density becomes far higher than the threshold of 250 to 300 mJ/cm^2^, narrow stimulated peaks grow sharper and higher, fluctuating randomly on the top of the emission band due to interference in disordered multiple scattering. Due to this light-in-light-out relation, in Figure 1c–f, the threshold can be roughly defined at 240, 255, 290, and 220 mJ/cm^2^ for RYGB random laser, respectively. To prove the nearly unchanged CRI with different output power intensities, the CRI under different pumping energies is computed in Figure 1b. As a result, the CRI of the white RL oscillates between 80 and 90, which promises both high color rendering quality and stability. The emission photograph of the white RL film is confirmed in the inset of Figures 1b and S1. The simulation of R9 values presented in Table S1 (Supporting Information) is also essential for white light applications. The R9 value in the spectrum quantifies its capability to render vibrant red hues accurately. An R9 rating is deemed suitable when it is around 80, which signifies superior color rendering for red tones, which is crucial for a range of applications. On the other hand, a lower R9 value means the light source may not accurately render intense red colors, leading to a weak representation of objects with significant red content. Most of the R9 values gathered in different pumping energy densities range from ∼70 to 85, which is reasonably good for a white RL. To the best of our knowledge, only a few reports have been published regarding white RLs,^20,26,27^ especially investigating their CRI and R9 values. Most of the previous studies found to calculate the CRI are from conventional lasers.^28,29^ While a recent paper introduced the simple fabrication of a pure and stable white RL,^20^ the investigations on the critical factors and physical mechanism of its superior characteristics to conventional laser have not been thoroughly evaluated. By using the four monochromatic polymer films, the result suggests that white RL can not only provide an almost uniform chromaticity, as shown in the previous study^20^ but also have good performance and a high CRI under different pumping energy, whether under or above the threshold. Because of this, the fabricated white RL promotes emission over large wavelength intervals, rendering it advantageous for applications that need a light source with a wide-ranging spectrum.

**Figure 2:**
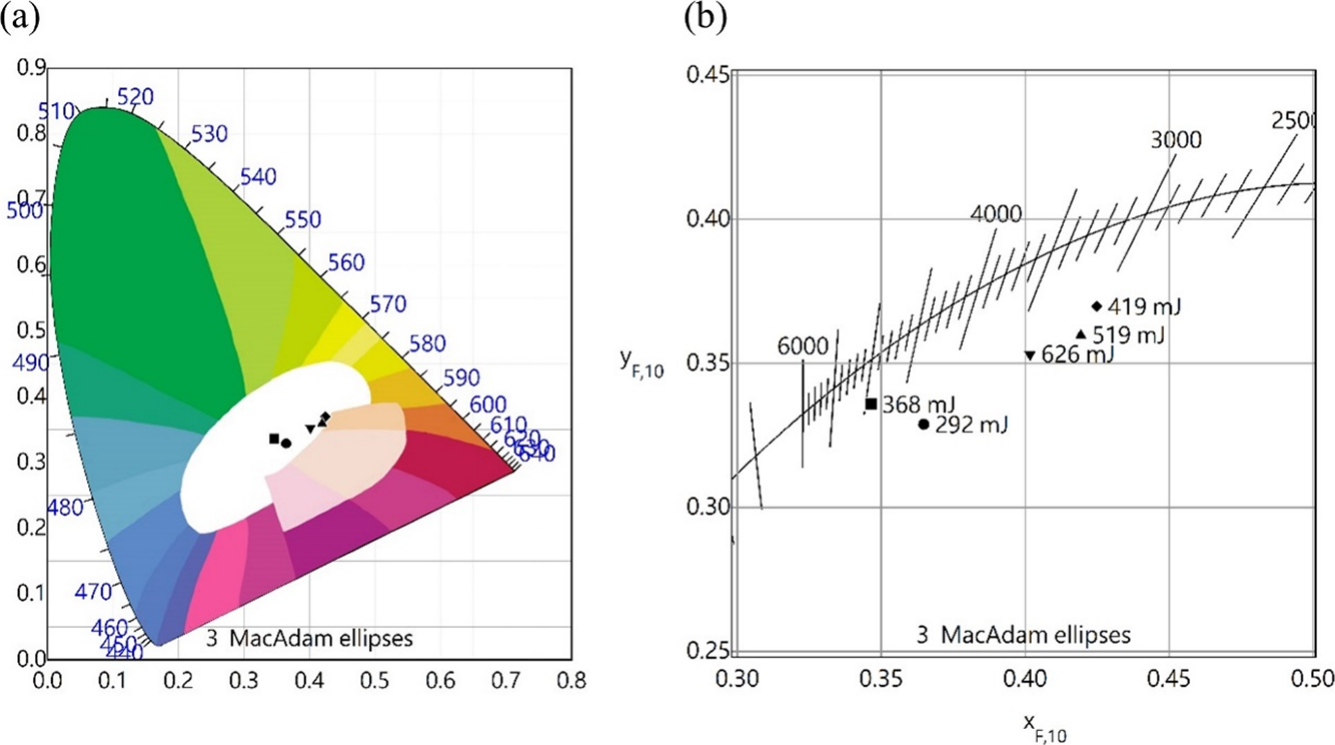
(a) CIE color coordinates with areas attributed to approximate colors and (b) CIE enlarged image under different excitation energy densities.

The CIE coordinates, as one of the indicators of a light source, are considered highly important to quantify the color of the white light sources, considering the eyes’ sensitivity to different colors. To determine the color of the visible emission of white RL that the human eye perceives, the CIE coordinates were calculated and are shown in Figure 2. Fortner et al. proposed assigning approximate colors to places on the CIE diagram to indicate the hue of the emission from general illumination sources.^30^ As shown in Figure 2a, the CIE coordinates of the light emitted by the white RL with different pumping energy densities are located at the same white area, confirming its feasibility of being an ideal white light source. Figure 2b shows the enlarged image of the CIE coordinates of the white RL with different pumping energy densities. The estimated *x*, *y* coordinates are found to be (0.418, 0.358), (0.412, 0.370), (0.418, 0.367), (0.416, 0.350), and (0.399, 0.349) corresponding to the 292, 368, 419, 519, and 626 mJ/cm^2^ excitations, respectively. When the pumping energy density is above the threshold (290 mJ/cm^2^), we can observe that as the pumping energy density increases, the CIE coordinates of the emitted white RL gradually shift toward the coordinates of ideal white light (0.33, 0.33). The color temperature, CCT, obtained from CIE coordinates, with a numerical number of about 3000 K, is categorized as “warm white” suitable for lighting bedrooms, living rooms, and restaurants.^31,32^

**Figure 3:**
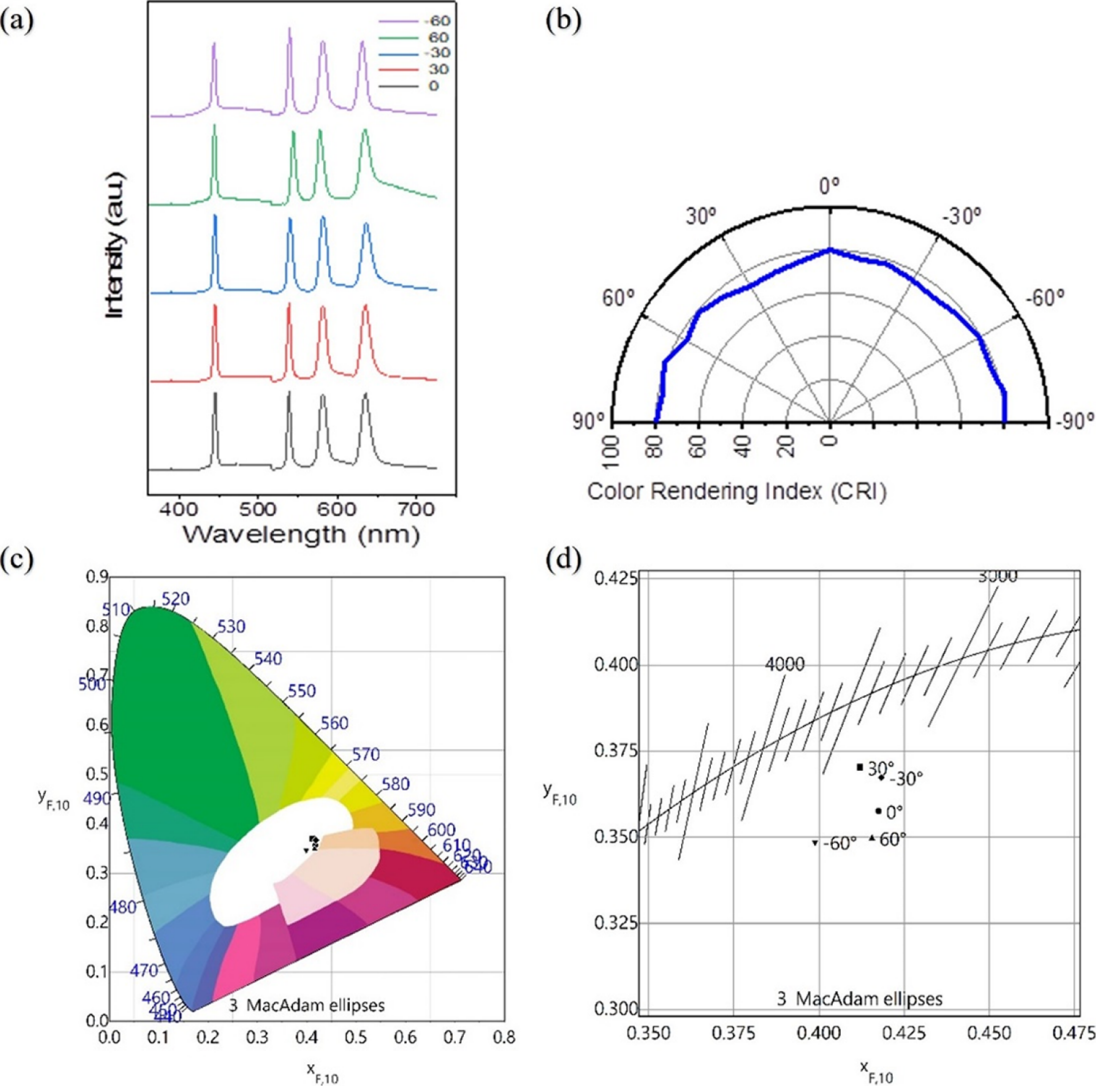
(a) White RL emission spectra. (b) CRI value of white RL under a broad-angle observation. (c) CIE color coordinates and (d) CIE enlarged image under a broad-angle observation.

Figure 3 shows the emission spectra and the CRI of white RL under broad-angle observation. With the full solid angle emission of 4π, an ideal light source should equally render the color at different angles, referring to an angle-free and affordable CRI. This motivates us to study the CRI of random laser at different angles since the lasing spectra of random laser can be observed from all directions. Due to the different laser cavities formed by multiple scattering providing broad-angle output directions, the angle-free light emission property makes white RL a born-to-be general lighting source, letting laser illumination devices free of the bulky, expensive, and complex optical devices. As shown in Figure 3a, the spectra of white RL were recorded with fixed pumping energy density (626 mJ/cm^2^) at various observation angles from θ = −90° to 90° (θ represents the angle formed by the normal vector of the sample and the direction of observation). Besides, the wavelength, the line width, and the intensity of the white RL emission are proved to be independent of the observation angle, and these behaviors exhibit a distinct indication of the occurrence of random laser activity. To reveal the angle-free CRI of white RL, the CRI values of white RL irradiation at wide observation angles have also been obtained, as shown in Figure 3b. As expected, the white RL can provide broad-angle light emission with an almost angle-independent CRI value of 80–85. One unique feature of random lasers is their ability to exhibit omnidirectional (multidirectional) emission, resulting from the light being scattered multiple times in disordered systems. This random laser property demonstrated in this study can be helpful for display technology and environmental lighting applications. Another characteristic suitable for display applications is the broad angular distribution of random laser even in a complete 4π solid angle which electrical control over laser emission is necessary.^33,34^ Since there were no noticeable changes and the white RL is flexible in emitting light over a wide range of angles, we believed that using symmetry can estimate its characteristics in a complete 4π solid angle. Also, a stable emission intensity independent of the observation angle (θ = −90° to 90°) was observed, verifying it can be a random laser with ideal white emission at multiple directions which can 4π solid angle. This verifies that white RL prevails over conventional laser with high directivity at more common illumination applications, proving the high color rendering quality from the full solid angle. Apart from investigating the CRI of the white RL, spatial emission chromaticity of the white RL is carefully studied by changing the detection angle θ relative to the substrate. Figure 3c shows the chromaticity of these spectra on a CIE 1931 color diagram. Figure 3d shows the CIE enlarged image under a broad-angle observation of fixed pumping energy density (626 mJ/cm^2^). As shown in the diagram, it has been confirmed that the emission chromaticity of white RLs is independent of the detecting angle. Based on the data presented in this article, we can confirm that the illumination quality of white RLs remains consistent regardless of the observation angle. This fully showcases the advantages of using random lasers for illumination and demonstrates the angle-free nature and full-angle color rendering ability, which are not yet found in conventional laser systems.

In order to better demonstrate the value of white RL as a light source, we considered using the CRI of white lasers constructed with traditional lasers as a reference for comparison. However, due to limitations in the available instrumentation, it would be quite challenging to construct white light with traditional lasers with peak positions identical to RYGB random lasers. Therefore, in the present work, we have simulated the spectrum of each laser as a single delta function of 1 nm bandwidth located at the center wavelength of its output, which is identical to the peak positions of the RYGB random laser. Figure S2 displays the white laser spectrum, which is comprised of a simulated spectrum consisting of four distinct colors. The wavelengths corresponding to the highest intensity points in the spectrum of the laser with four colors are 446, 539, 583, and 635 nm. The computed spectrum displays peak positions and intensities that align with the excitation of white RL at 626 mJ/cm^2^. It has been observed that the spectra exhibit nearly identical lasing characteristics. After gathering the spectrum of the laser with four colors, we simulated its CRI by normalizing each spectrum and importing the data in the commercial software for LEDs (Color Calculator, Osram Sylvania Inc.) to calculate and gather the laser characteristics such as CRI. By performing this, the CRI that we gathered for the simulated four-color laser spectrum is 61, which is considerably lower than the CRI of our experimental white RL (CRI: 82). This suggests that random lasers have the potential to offer a higher CRI compared with conventional lasers that emit multiple wavelengths. The high CRI value of white RL can be attributed to the distinctive emission spectra of random lasing systems. As shown in Figure S3, several sharp peaks with line widths less than 1 nm emerge on the top of the emission band when the pumping energy is above the threshold. Manifold narrow spikes form from the multiple scattering of light that occurs among the randomly distributed Ag nanoparticles. Unlike the spectrum of traditional lasers, the multiple sharp peaks observed in the emission spectrum of white RLs can be considered as the combination of multiple close but distinct laser lights, thus contributing to the high CRI of the white RL. In conclusion, benefiting from the multiple spikes in the spectrum caused by multiple scattering, the white RL covers almost the entire visible spectrum, demonstrating a high CRI value that is lacking in traditional lasers.

**Figure 4:**
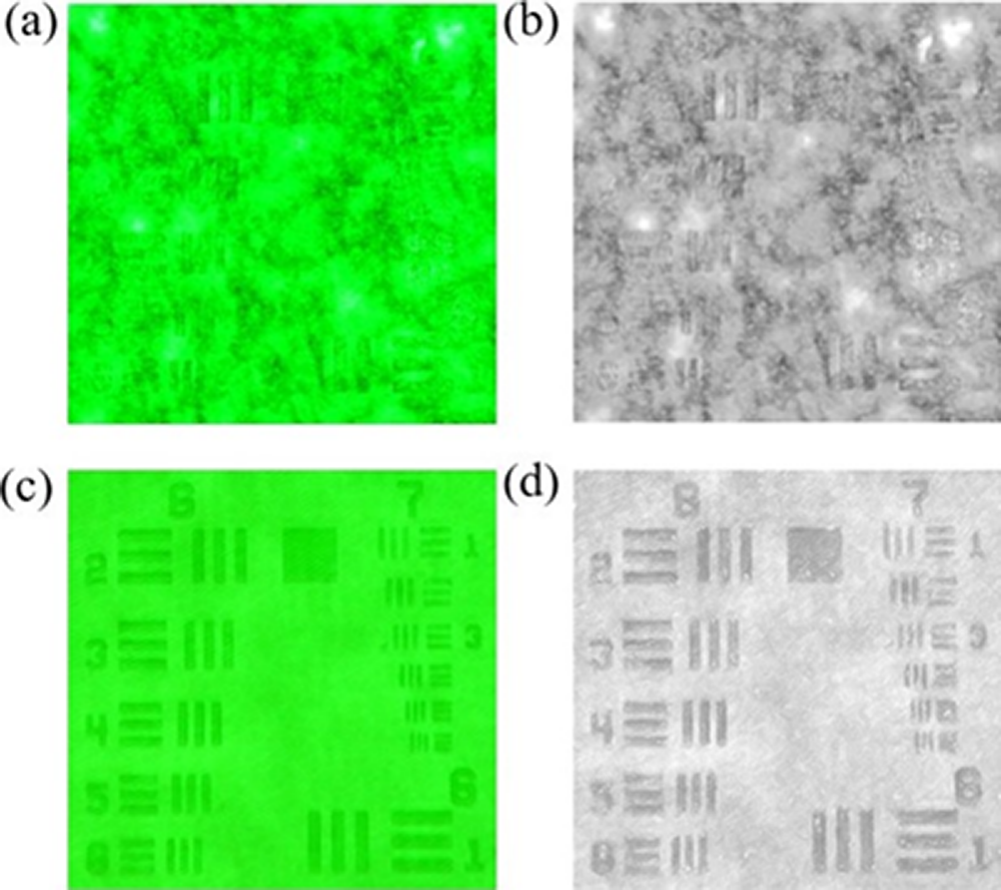
AF chart images taken with conventional laser and random laser light sources. (a, b) 532 nm conventional laser images on AF chart and (c, d) green random laser images on AF chart.

Figure 4 displays the AF chart photographs captured under the illumination of a 532 nm conventional laser and the green random laser. The photographs gathered clearly demonstrate that the random laser effectively inhibits the formation of speckle noise. As shown in Figure 4a,b, for conventional lasers, inevitable speckle noise caused by high coherence becomes a challenge in the application of illumination. In contrast, as shown in Figure 4c,d, precluded interference leads to the deduction of speckles, and the speckle-free imaging using a random laser was obtained when illuminated with a low-coherence light source.^17,35,36^ To reproduce the experimental result in the previous study^36^ and remark on the potential of white RL for general lighting, we compared images generated with green random laser illumination to those generated by other common light sources (e.g., 532 nm conventional laser) on the 1951 US Air Force (AF) resolution test chart. As a result, the image under white RL illumination exhibits not only high color rendered as shown in Figure 4c,d but is also speckle-free. The results can be one of the direct solutions to the real challenge of laser speckle in laser lighting.

Based on the results gathered, the white RL outperforms conventional lasers in certain aspects, making it a promising alternative for various full-field imaging applications. Also, several important factors and physical mechanisms can enable the white RL to achieve its high-brightness illumination. The random laser medium is fundamental in recognizing a specified spectrum of emitted light. It needs to provide sufficient optical gain to surpass losses and reach the lasing threshold.^37^ This condition was achieved as shown in Figure 1c–f, wherein the optical gain became stronger than the loss when the power density increased near the threshold. This process stimulated the emission and resulted in the emergence of RYGB color. The RYGB spectra were measured from four organic lasers (monochromatic polymer films) light-pumped under various energies. Due to the application of different pumping energy densities, stimulated peaks randomly fluctuate on top of the emission band due to interference caused by disordered multiple scattering, thereby raising the amplification efficiency of light. While conventional lasers emit light that is highly directional, random lasers have more diffused emission and operate based on multiple scattering in a disordered medium rather than using conventional laser cavity mirrors for feedback.^38^ The strong interference effects due to multiple scattering can provide coherent feedback, which leads to laser-like phenomena of threshold behavior and spectral narrowing, as shown in Figure 1.

Conventional lasers and superluminescent LEDs (SLEDs) are highly luminous. However, they are not appropriate for full-field imaging applications due to their high spatial coherence, resulting in speckles (coherent artifacts) that can diminish the image quality. On the other hand, random lasers exhibit low spatial and temporal coherence, which is advantageous to enhance the image quality and broaden its applications in full-field and speckle-free imaging.^17,20,34^ This was verified as shown in Figure 4a,b, wherein the white RL fabricated in this study was used as a light source, and after passing through the AF chart, no speckles were generated. Compared with conventional lasers, the white RL covers nearly the whole visible spectrum, indicating a high and unchanged CRI value with different output power intensities, showcasing its high color rendering quality and stability. Furthermore, white RLs can produce emissions within a broad angular range, a feature not seen in conventional and traditional lasers. The chromatic coordinates of white RL do not show substantial changes when observed from different angles, as described in Figure 3a–d. White RLs can possess the capability to color tunable lasing, providing flexibility in the emitted light, which is important for applications requiring adjustable illumination.^20^ Other characteristics of random lasers include superior imaging quality in complex scattering environments, high photon degeneracy or spectral radiance, and versatility in pumping methods.^17,39^ These results demonstrate that the white RL in this study offers high-brightness illumination and exhibits superior performance compared to conventional lasers and SLEDs, making them appropriate for applications requiring strong-luminous, speckle-free, and high-efficiency lighting. To further enhance the intensity of the fabricated random lasers, there exist several plausible ways, such as using highly efficient emission of quantum dots, metal nanoparticles, and hyperbolic meta-materials as shown in published reports.^26^

## 4 Conclusions

In this study, we have successfully demonstrated a white RL composed of RYGB random lasing devices. The white RL exhibits a stable and sufficiently high CRI value under various pumping energy densities. Furthermore, by analyzing the CIE chromaticity coordinates of white RL, we have shown that the CIE coordinates of white RL do exhibit moderate variations with increasing pump energy density. The result indicates that this high-CRI light source retains a warm white color hue as the pumping energy density increases. Unlike traditional lasers, random lasers exhibit an angle-free emission characteristic due to the multiple scattering in disordered nanostructures. By investigating the white RL spectra at different viewing angles, we have demonstrated that white RL emits light with angle-independent and sufficiently high CRI values. Furthermore, the chromatic coordinates of white RL do not exhibit significant variations at different observation angles. This indicates that the emission color of white RL remains consistent irrespective of the viewing angle. To provide a more rigorous comparison of the differences in light source quality between traditional lasers and white RL, we simulated an RYGB laser that corresponds to the four peak wavelengths of white RL. The result shows that the white RL covers almost the entire visible spectrum, demonstrating a high CRI value that is lacking in traditional lasers. In this study, white RL and traditional lasers were used as light sources, and their imaging on a USAF chart after passing through a scattering film was compared, as demonstrated in our research. The result sufficiently demonstrated that random lasers, due to their inherent characteristics, do not generate speckles when applied for illumination. In conclusion, we have systematically compared several optical properties between white RL and traditional lasers, and these data provide compelling evidence that white RL indeed holds higher potential as a light source for illumination when compared to traditional lasers. Notably, electrically driven white RL is an excellent research topic for future study, which may be achievable by following the methodology as shown in our previous work.^40^

## Supporting Information Available

Photograph of the white random laser (white RL) film; lasing spectra of the random laser; spectrum of four colors simulated conventional laser; schematic representation of the experimental setup for the images of the AF chart taken with conventional laser and random laser light sources; and R9 values in different pumping energy densities of the white random laser (PDF)
