# Supplementary material for: Speckle-Free, Angle-Free, Cavity-Free White Laser with a High Color Rendering Index
Source: ACS Appl Mater Interfaces. 2024 Feb 23;16(9):11489–96. doi: 10.1021/acsami.3c17222 (PMC10921373; doi:10.1021/acsami.3c17222)
Supplement: Supplementary file 1 — am3c17222_si_001.pdf [file am3c17222_si_001.pdf]

Supporting Information

Speckle-free, Angle-free, Cavity-free White Laser with High Color Rendering Index

Cheng-Fu Hou[a,†], Wei-An Tsui[b,†] , Rou-Jun Chou[a,†] , Chih-Hao Hsu[b] , Denice N. Feria[b], Tai-Yuan, Lin[b,] , Yang-Fang, Chen[a,*]*

[a] *Department of Physics, National Taiwan University, Taipei 10617, Taiwan*

[b] *Department of Optoelectronics and Materials Technology, National Taiwan Ocean University, Keelung City 202301, Taiwan*

[†] *These authors contributed equally to this work and should be considered as co-first authors.*

*yfchen@phys.ntu.edu.tw

*tylin@mail.ntou.edu.tw

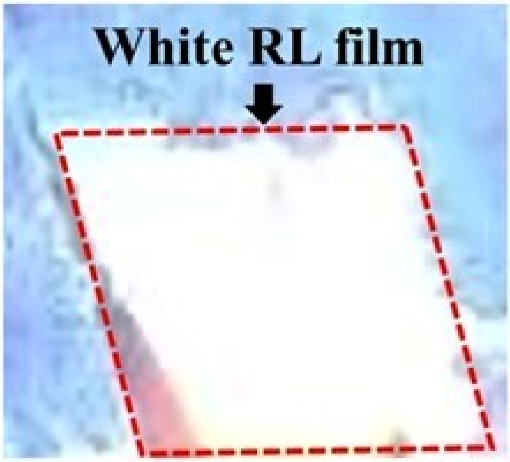

Figure S1. Photograph of the white random laser (White-RL) film.

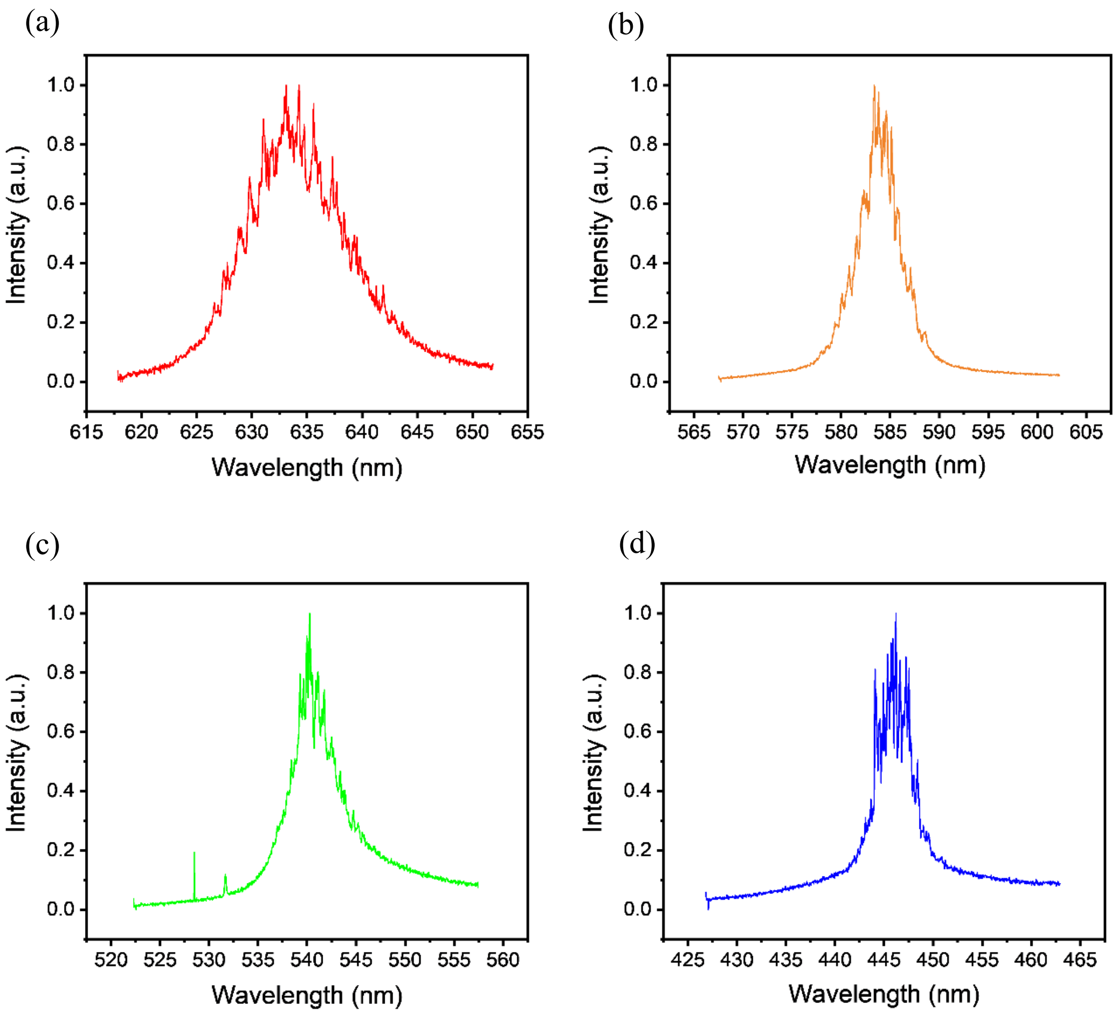

Figure S2. Lasing spectra of (a) red, (b) yellow, (c) green, and (d) blue random laser.

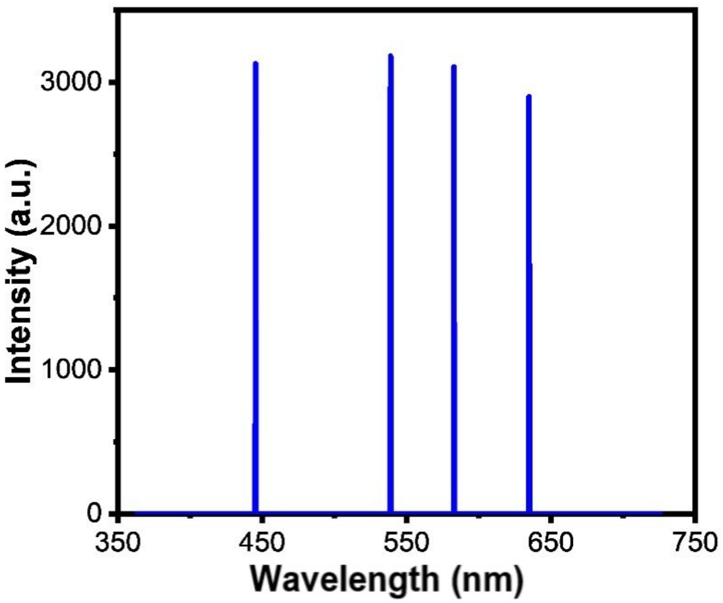

Figure S3. Spectrum of four colors simulated conventional laser.

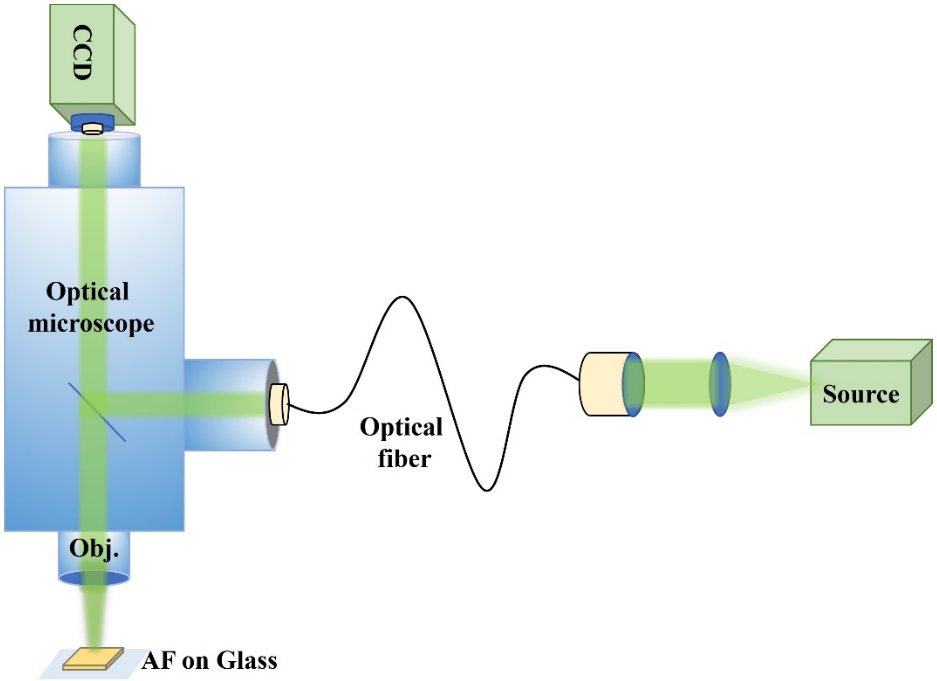

Figure S4. Schematic representation of the experimental set-up for the images of the AF chart taken with conventional laser and random laser light sources.

Pumping Energy Density	R9 values
292 mJ/cm^2	72
368 mJ/cm^2	37
419 mJ/cm^2	85
519 mJ/cm^2	80
625 mJ/cm^2	79

Table S1. R9 values in different pumping energy densities of the White Random Laser.
